# Peeking into the future: inferring mechanics in dynamical tissues

**DOI:** 10.1042/BST20230225

**Published:** 2024-12-10

**Authors:** Augusto Borges, Osvaldo Chara

**Affiliations:** 1 Unit Sensory Biology and Organogenesis, Helmholtz Zentrum München, Munich, Germany; 2 Graduate School of Quantitative Biosciences, Ludwig Maximilian University, Munich, Germany; 3 School of Biosciences, University of Nottingham, Sutton Bonington Campus, Nottingham LE12, U.K.; 4 Instituto de Tecnología, Universidad Argentina de la Empresa, Buenos Aires, Argentina

**Keywords:** development, mechanobiology, morphogenesis, stress inference

## Abstract

Cells exert forces on each other and their environment, shaping the tissue. The resulting mechanical stresses can be determined experimentally or estimated computationally using stress inference methods. Over the years, mechanical stress inference has become a non-invasive, low-cost computational method for estimating the relative intercellular stresses and intracellular pressures of tissues. This mini-review introduces and compares the static and dynamic modalities of stress inference, considering their advantages and limitations. To date, most software has focused on static inference, which requires only a single microscopy image as input. Although applicable in quasi-equilibrium states, this approach neglects the influence that cell rearrangements might have on the inference. In contrast, dynamic stress inference relies on a time series of microscopy images to estimate stresses and pressures. Here, we discuss both static and dynamic mechanical stress inference in terms of their physical, mathematical, and computational foundations and then outline what we believe are promising avenues for in silico inference of the mechanical states of tissues.

## Introduction

Tissue morphogenesis is driven by changes in cell numbers (in turn caused by mitoses, apoptosis, and cell extrusion), collective movements of cells, and changes in cellular mechanical properties together with alterations of the constraints imposed by the environment onto the epithelium [1–10]. Hence, tissue mechanics needs to be addressed to understand the remarkable morphogenetic processes that shape embryonic tissues during development and the outgrowth of tissues in species capable of regeneration [11].

**Figure 1.**
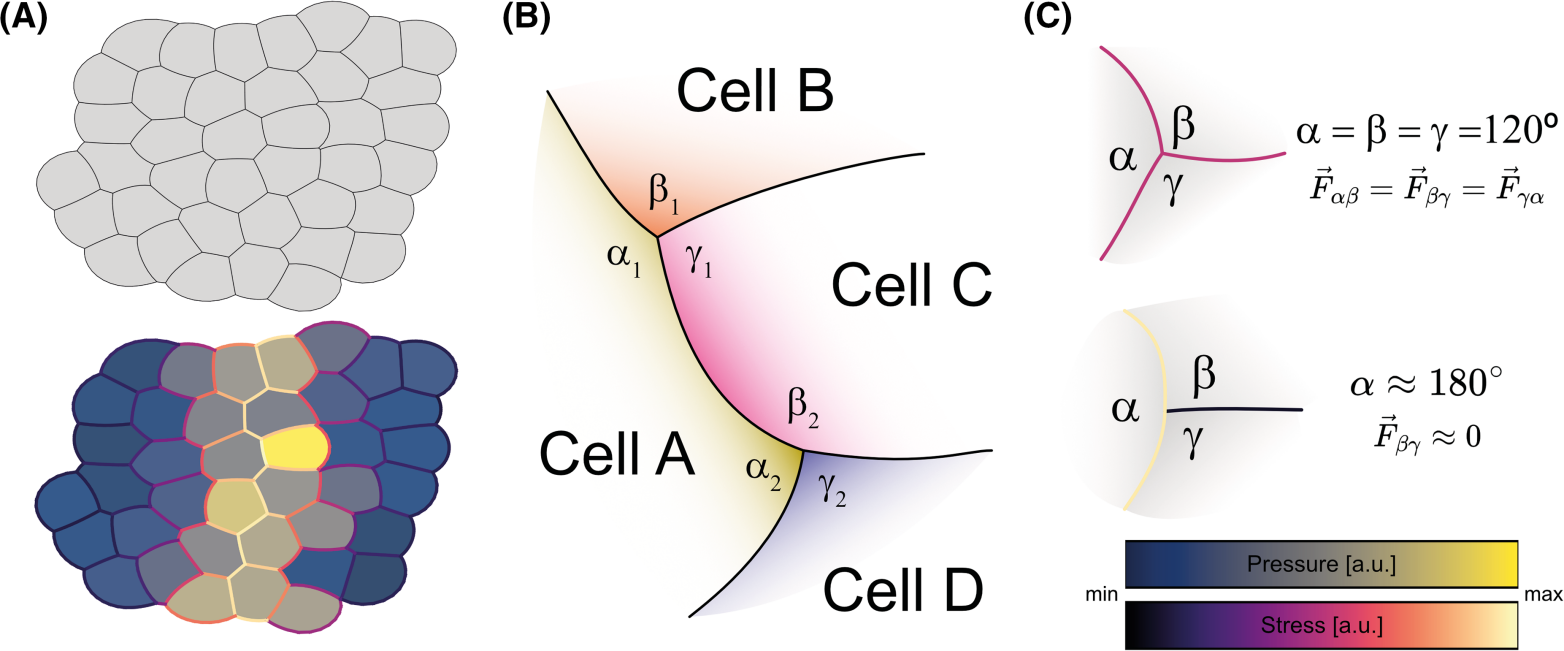
Stress inference, conceptually. (**A**) Stress inference uses the shape of the cells in a tissue (shown in A, upper) to estimate the stress acting on them (color code shown in C, lower). The tissue model was generated using Surface Evolver [107] through the seapipy software, generating a high-stress furrow in the center. Stress maps were created using ForSys software [66]. (**B**) Cells A and C share a common edge between triple junction 1 and 2. At each of these junctions, the incoming membranes have a contact angle $\alpha_{1}$, $\beta_{1}$, and $\gamma_{1}$ and $\alpha_{1}$, $\beta_{1}$, $\gamma_{1}$ for the mentioned junctions 1 and 2, respectively. (**C**) Contact angles determine the relative tension of each membrane. For equal angles (upper panel), the tensions will be similar. If an angle is a straight angle, the other membrane will have a tension of zero. The color map represents the stress and pressure for panels A and C in a relative scale from minimum to maximum. Pressure values in the lower part of panel A have transparency to distinguish them better from the stresses.

The study of forces in embryo morphogenesis can be traced back to the *Entwicklungsmechanik* (developmental mechanics movement), which emerged during the 19th century [12]. Later, D'Arcy Thompson enlightened his time with a revolutionary concept: the size and shape of body organisms could also be interpreted as a map of the acting and driving mechanical forces [13], in the same way in which Faraday conceptualized that iron particles in his experiments were a map of the invisible magnetic fields underneath [14] (Supplementary Figure S1A–C). Thus, D'Arcy Thompson transformed our way of perceiving living organisms: The geometrical features of organisms, tissues, and cells reflect not only gene regulatory networks but also a vivid manifestation of mechanical forces at work (Figure 1A). This idea re-emerged and crystallized into a mathematical method to infer mechanical stresses in tissues during the late part of the last century.

Tissue shape is not solely a product of biochemical signaling, but results from the collective interactions between constituent cells [15–19]. Changes in the mechanical interactions between cells and their environment/substrate, reflected in tissue stiffness, can promote epithelial-mesenchymal transitions, which promote tissue repair and development [20–23]. In tumorigenesis, increased stiffness can lead to tumor progression and metastasis [21,24,25]. Spatial and temporal changes in stiffness can influence the cell-to-cell stresses that arise in tissues. Interestingly, tissue stress distribution is not temporally invariant during complex biological processes such as development [26–28]. For example, tissue stress fluctuations have been shown to play a key role in facilitating tissue reorganization in the developing zebrafish [29]. Mechanical stress also plays a critical role in tissue regeneration by promoting cell functions such as proliferation, differentiation and migration [30–32]. Mechanical stimuli can improve bone fracture healing by promoting the growth and differentiation of bone-forming cells [30].

This mini-review^1^ first describes the computational methods of static stress inference, their advantages over the standard experimental stress determination, and their limitations. Next, we discuss how these limitations lead to the alternative computational methods of dynamic stress inference, which essentially use the topology of the tissue in the ‘present’ combined with its ‘future’ state to predict the stresses therein. We explore the similarities and differences between the dynamic and static counterparts and highlight their advantages and disadvantages. We have summarized all stress inference algorithms that exist to the best of our knowledge in Table 1. We conclude this review with what we believe are exciting future perspectives of stress inference in tissues.

**Table 1.** Existing stress inference methods.

Method	Static/dynamic	2D/3D	Algorithm	The biological system to which it was initially applied to	Software availability	Ref. and year
VFM	Dynamic	2D	Finite element mesh; Straight edges; Stress/pressure together; Least squares solver	Ventral furrow formation in Drosophila, *in vivo*	Not available	[57], 2010
Chiou et al.	Static	2D	Straight edges; Stress/pressure together; Inverse solution	Ventral furrow formation in Drosophila, *in vivo*	Not available	[39], 2012
Bayesian	Static	2D	Straight edges; Stress/pressure together; Bayesian solver	Drosophila pupal wing, *in vivo*	Open source on GitHub	[59], 2012
CellFIT	Static	2D	Curved edges; Stress/pressures separated; Least squares solver	Dorsal closure and imaginal disk in Drosophila, *in vivo*; Dragonfly wing	Not available. It can be found as a binary file	[60], 2014
CellFIT-3D	Static	3D	Curved edges; Only stress; Least squares solver	Murine embryos	Not available	[98], 2017
DLITE	Static*	2D	Curved edges; Stress/pressure separated; Least square solver	Human stem cell colonies, *in vitro*	Open source on GitHub	[67], 2019
VMSI	Static	2D	Fitted curved edges; Stress/pressure separated; Variational solver	Drosophila embryogenesis, *in vivo*	Implemented by Hallou et al. [103]	[40], 2020
foambryo	Static	3D	Curved edges; Stress/pressure separated; Least square solver	Ascidian embryo *Phallusia mammillata*; *Caenorhabditis elegans* embryo	Open source on GitHub	[92], 2023
ForSys	Dynamic and Static	2D	Curved edges; Stress/pressure separated; Least-square solver	Zebrafish Lateral Line, *in vivo*; Xenopus Mucociliary epithelium, *in vivo*	Open source on GitHub	[66], 2024

Details all currently existing stress inference methods, whether dynamic and/or static, 2D or 3D support and their algorithm, along with their original application and current availability.

## Determining stress statically: the geometrical stress inference

Computational estimation of the mechanical forces at play in biological systems was originally called Force inference, even though the calculated magnitudes are scalars. Here, as pointed out by other authors [35,39,40], we will use the term stress inference. This inexpensive technique approximates the relative stresses and pressures operating in a given system using a microscopy image as sole input, exploiting the information in the tissue topology to obtain the apparent distribution of stresses therein [35,41–46].

The contact angle among cells can serve as a proxy for cell-cell junctional tension, a concept imported from wetting phenomena to study cell interfaces [47] (Figure 1B). Thus, it can be used for stress determination between cells in monolayers, as it depends on the relative forces along the interfaces [48,49]. The cell-cell junctional tension might arise from different sources, such as actomyosin contraction due to myosin accumulation at the cortex or cell-cell adhesion through cadherin binding [2,50]. The membrane stress will be equal at a tricellular junction where all cells meet with the same contact angle ($\alpha =\beta =\gamma =120^{\circ }$) (Figure 1C, upper panel). On the other hand, when there is a near-straight angle between two membranes, the stress in the third one will be almost zero (Figure 1C, lower panel). As determining the contact angles at each junction is paramount, membrane segmentation needs to be precise to avoid angle misrepresentation. New image segmentation software is appearing [51–53], and constant efforts are being made to standardize their applicability [54].

**Figure 2.**
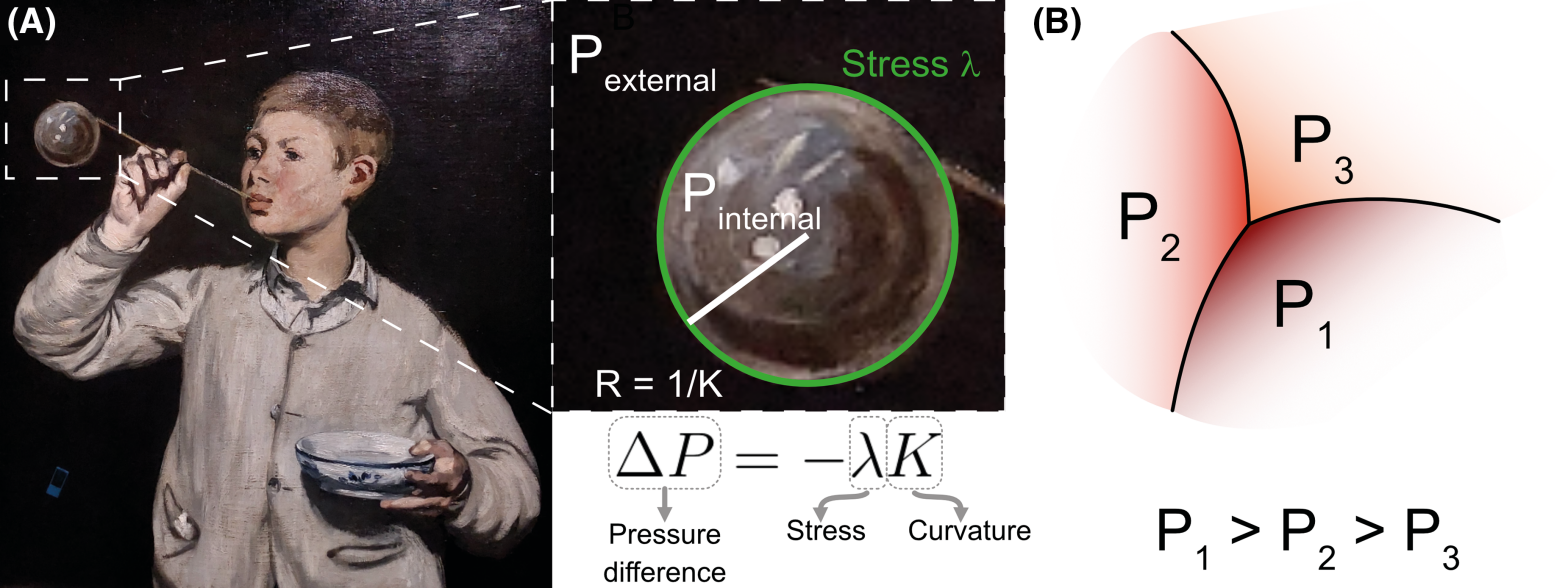
Stress and pressure are determined by shape. (**A**) The pressure on the soap bubble of the ‘Les Bulles de savon’ (1867), Édouard Manet, Calouste Gulbenkian Museum, Lisbon (Picture acquired by OC in the exposition Manet/Degas au musée d'Orsay), can be determined by the Young-Laplace equation, relating pressure difference (Δ*P* = *P*_internal_  −  *P*_external_) with stress on the surface (λ) and curvature of its shape (*K* = 1/*R*). (**B**) Scheme representing pressure differences for a given geometry. Cells with higher pressure will be more convex, while lower-pressure cells will tend to be more concave. The color intensity represents pressure values qualitatively on a relative scale from lower (orange) to larger values (red).

Therewith, the intracellular pressure can be inferred using the Young-Laplace Law (Figure 2A) by combining the intercellular stress and the local cell membrane curvature. Conceptually, more convex cells will have a higher pressure than concave ones (Figure 2B). Pressure differences arise from an interplay between the hydrostatic pressure in the cell's environment and the osmotic pressure due to the cell's semipermeable membrane. Only recently have the osmotic contributions to the intracellular pressure been determined *in vivo* in zebrafish [55].

Static stress inference requires the tissue of interest to be in mechanical equilibrium, allowing each cellular junction to be associated with a force balance equation. Using Newton's second law, each equation will have the sum of forces acting on a given vertex equal to zero,

1 $$\underset{j}{\sum }\vec{F_{j}}=0$$

where *j* identifies the different edges in the junction. The absence of inertial terms is justified by assuming that the system behaves as a viscous fluid. Thus, these components are negligible compared with viscous ones [56].

The precise mathematical form of the forces at each edge must also be decided. The most frequent choice is to consider the force in the direction of the edge scaled by its stress [57–60], similar to what is done in vertex models [39,44,61–63]. The force at each membrane would be associated with the vectorial equation:

2 $${\vec{F}}_{j}=\lambda_{j}{\vec{r^{i}}}_{j}$$

Here, $\lambda_{j}$ represents the stress of membrane *j*, ${\vec{r^{i}}}_{j}$ the unit vector in the direction of the edge *j* from junction *i*. Importantly, although choosing a model for the stress on the edge is necessary, it is not a unique choice [58].

## Determining edge shape and estimating pressure

**Figure 3.**
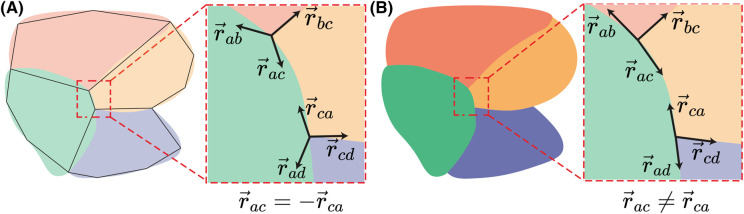
Approximating the shape of the membrane. (**A**) If cells are polygonal, cell membranes can be approximated in the system with straight lines joining the junctions, seen here as ${\vec{r}}_{ab}$, ${\vec{r}}_{bc}$, ${\vec{r}}_{ac}$, and ${\vec{r}}_{ca}$, ${\vec{r}}_{ad}$, ${\vec{r}}_{cd}$. In this situation, the vector joining two junctions will have the same magnitude but opposite sign at each junction. (**B**) In contrast, curved edges give a better approximation of the shape and more stability to the system of equations, as a curved shape adds more independent information to the geometric determination. Note that vectors ${\vec{r}}_{ac}$ and ${\vec{r}}_{ca}$ are no longer collinear in a curved shape approximation.

The proper determination of the membrane's shape is crucial for stress inference. Noisy images might lead to numerical errors that get amplified down the pipeline, reducing the accuracy of the inference. Several methods can be used to estimate the membrane's shape contribution to the geometrical matrix defined in eqn 2. Straight edges, though easy to estimate from microscopy images, have the inconvenience of making the system less robust [35,60]. This can be visualized, for example, by taking an edge and realizing that the force will have the same components at each end, with an opposite sign (Figure 3A). On the contrary, curved edges will have a distinct value at each end of the edge, adding independent information to the system of equations (Figure 3B). In this case, each edge's direction (vector) could be determined from the tangent to the limiting angle to the cell membrane [60].

Early methods inferred cortex stresses and cell pressures simultaneously and used the straight-edge approximation [35,57,58]. These methods assume that the cell's mechanical energy will depend only on the length of the edges and the cell's area to derive the system of equations from a potential energy [39,59]. Having the pressure and stress intermixed in the same expression increases the number of unknowns per equation while maintaining only one equation for each space component. Moreover, tissue boundaries and fourfold vertices give fewer equations than unknowns, leading to an underdetermined system. Due to the constraints mentioned, this type of system has been solved using Bayesian methods [19,45,59]. These constraints are not unavoidable, as was demonstrated by the Cellular Force Inference Toolkit: CellFIT [60]. This method allows considering curved edges and makes stress and pressure inference independent. Pressure is modeled using the Young-Laplace equation. This equation relates the shape and stress of an interface with the difference in pressure between both sides (Figure 2A). As the stress in an edge is required to calculate the pressure, this inference needs to be performed subsequently, effectively decoupling both magnitudes.

## Finding the solution to the inference problem

**Figure 4.**
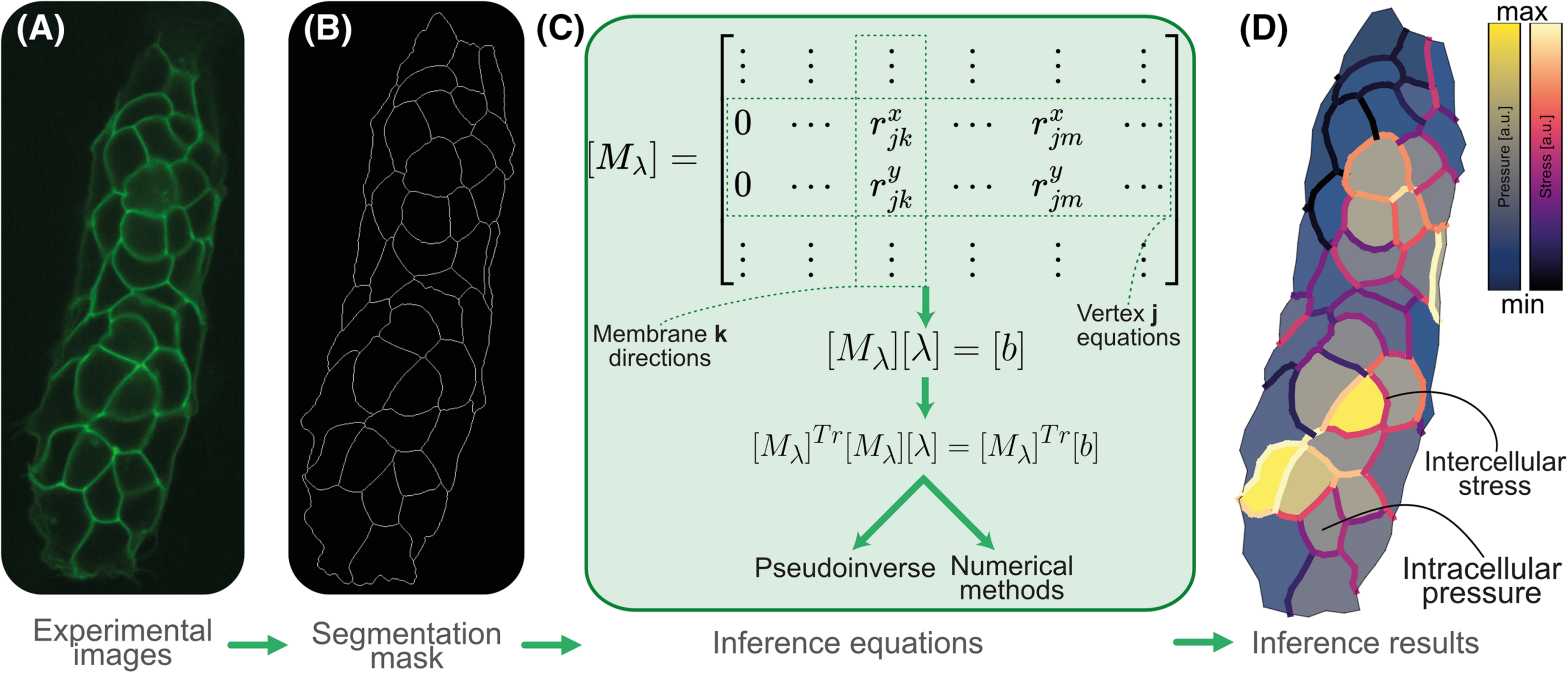
General stress inference pipeline. Starting from an experimental image, such as a primordium in the Zebrafish lateral line (**A**), a segmentation mask has to be generated (**B**) to construct then a Geometrical Matrix encompassing the information about the shape of cells, as described in the section ‘Finding the solution to the inference problem’ (**C**). The solution can be found by inverting the system, using a pseudoinverse, or using different numerical methods. Intercellular stresses are inferred from these equations (**D**). The intracellular pressures are inferred using the Young-Laplace equation (see Figure 2A). The primordium image in panel (**A**) was taken with a spinning disk confocal microscopy. The green fluorescence marks cell membranes in green. The primordium membranes shown in panel A were tagged using claudnb:lyn-EGFP. Stress inference was executed by using ForSys [66]. Pressure values in panel D have transparency to distinguish them better from the stresses.

Each microscopy image (Figure 4A) is segmented (Figure 4B) and then converted to a system of equations (Figure 4C) when performing stress inference (Figure 4D). Each spatial dimension will contribute an equation per junction and one unknown per membrane: the stress. The most common case is to have triple junctions, i.e. junctions with three connecting membranes [64,65] (Figure 1B). In two dimensions, each new junction will contribute two equations and, at most, three unknowns, as some membrane stresses will be repeated.

This is commonly written in matrix form as

3 $$\left[M_{\lambda }][\lambda ]=[b\right]$$

In the following sections, quantities that appear in brackets [] are to be interpreted as matrices. $\left[M_{\lambda }\right]$ in eqn 3 summarizes all the geometrical information of a tissue, as it contains all components of the versors and their relationship to one another. Each column represents edge stress, and each row is the equation at a junction for one of the spatial co-ordinates (*x* and *y* in the 2D case). We call this matrix the *geometrical matrix* of the system since it reflects all the geometrical features of the edges connecting the junctions of the tissue under study (Figure 4C).

In geometrical stress inference, where no movement of the junctions is considered, the right-hand side of eqn 3 is set to the null matrix, i.e. [b]=0. If all the junctions are at rest, a possible solution to the system of equations would be that no stresses are acting on the system ($\lambda_{j}=0\;\forall j)$. Two methods are prevalent in the literature to avoid this unrealistic and trivial solution. We and other authors have included a constraint linking all the stresses in the tissue, such as a particular value for the average stress [57,58,60,66]. Fixing a value has the advantage of adding one more equation to the system without any new unknowns, thus increasing its stability. Another method consists of changing the cost function associated with the method, adding a regularizer [67] that penalizes the null solution. More generally, this could allow tailoring of the cost function to the specific needs of the problem at hand by enabling the addition of new terms, such as penalizing wider distributions of stresses.

Stress inference methods do not need to make any assumptions about the specifics of the force generation mechanism. However, stress inference may yield negative results if the inference method allows it or null stresses if not. In static stress inference, this usually means that the particular shape of a membrane is incompatible with the underlying model. Sometimes, it is possible to identify these issues and avoid problematic junctions or membranes. Typical pathological junctions include near right angles, curvy membranes, or junctions higher than three-fold [35,68,69].

Once stress is inferred, pressure can be determined by the curvature of the cell's membrane, as stated in Young-Laplace Law (Figure 2A). The equation can be made more explicit as

4 $$P_{j}-P_{i}=\lambda K$$

Here $P_{j}$ and $P_{i}$ are the pressures of cells *j* and *i*, respectively, λ is the membrane's stress, and *K* is the curvature. Therefore, an inhomogeneous system of equations can be assembled where the unknowns are each of the $P_{i}$, in matrix form

5 $$\left[M_{P}]\;\left[P\right]=\;[b_{P}\right]$$

Each row of the matrix $\left[M_{P}\right]$ represents an interface between two cells, with a 1 at the site of the first cell and *a* −1 at the site of the second cell, and each column relates to one of the rows in the [*P*] vector of unknowns. The matrix $\left[b_{P}\right]$ has the corresponding curvature of the membrane and its stress in each row. As with the stresses, an additional equation is commonly incorporated as a Lagrange multiplier to set a relationship between the pressures, usually making the sum of the pressures equal zero [66,69].

A system is said to be overdetermined when there are more equations than unknowns, meaning there is no exact solution in almost all cases. Generally, stress inference pipelines encounter overdetermined systems, with some exceptions [35,59]. A popular method to address this is to find an approximate solution through the Least Squares Method, which works by creating a new system of equations using the transpose of the matrix as

6 $$\left[M_{\lambda }{]}^{Tr}[M_{\lambda }][\lambda ]\;=\;[M_{\lambda }{]}^{Tr}[b\right]$$

and then inverting the new square matrix $\left[M_{\lambda }{]}^{Tr}[M_{\lambda }\right]$. The approximate (least squares) solution to the problem is then found by minimizing the difference between both sides of the equation using a Non-Negative Least Squares or a Least Squares solver [70–72]. An alternative method to solve the system of equations is to use the Moore-Penrose pseudoinverse [39,73,74]. The generalized inverse of a matrix [*A*] is defined as

7 $$[A{]}^{\text{‡}}=\;([A{]}^{Tr}[A]{)}^{-1}A^{Tr}$$

Then, given a system of equations as represented by eqn 3, the stresses would be expressed as

8 $$\left[\lambda ]=\;({\left[M_{\lambda }\right]}^{Tr}[M_{\lambda }]{)}^{-1}[M_{\lambda }{]}^{Tr}[b\right]$$

The mechanical inference pipeline is finally built as one system of equations coupling stresses and pressures, or two when decoupled, with the stresses and pressures as unknowns. Each system is built and solved for a single microscopy image (Figure 4D). However, if a specific problem has a time series of images, how can we incorporate the information about its evolution?

## Dynamic stress inference

Tissues in a quasistatic regime may be encountered during adult tissue homeostasis or late embryonic development. However, tissues are often found in a more dynamic state in early embryonic development where cell motility and rearrangements cannot be neglected [75,76]. During development, morphogenetic flows shape the organisms into functional forms, such as in Drosophila [77–80] and Zebrafish [81–85]. Therefore, accurate prediction of mechanical forces in these tissues requires inference algorithms that work optimally in the presence of significant motion. This modality of stress inference is called Dynamic Stress Inference [35].

A key challenge in Dynamic Stress Inference is to follow the tissue's evolution reliably through time, yet not all tissue elements have to be tracked. The elements can be divided into two categories: passive elements, which dissipate energy, and active elements, which generate work [57,58]. Brodland and colleagues suggest that the forces generated by active elements, such as the actomyosin network and cell membranes, deform the passive elements, including the cytoplasm, organelles, and extracellular matrix [58]. The inference method requires selecting an optimal level of detail, as the relevant elements need not only be the cellular cortex but could also be subcellular structures [57,58]. The selected structures must be defined in detail in the microscopy to allow tracking throughout the experiment.

The second challenge is also found in static inference and was mentioned above: the need to choose an underlying model for membrane stress. The choice depends on the specifics of the system of interest and the desired level of complexity. All currently available stress inference software uses a derived version of the model described by eqn 2. As written, this equation reflects the mechanical stress along the membrane, which is conceptually similar to the line tension of a vertex model [63]. However, in systems where the contractility of the actomyosin cortex is particularly relevant, an additional term may need to be added to model the stress along the entire circumference of a cell, such as the perimeter term of a vertex model [63].

Finally, the third key challenge relates to the question of the scales involved in the processes studied. In Static Inference, only the spatial scale is relevant, as it relates to the positions of the membranes among themselves and is ultimately used to find the angles. Importantly, in Dynamic Inference, the time scale comes into play, which implies that, in this formalism, the relation between scales of space and time affects the inference.

In summary, to successfully incorporate cell movement information into the inference process, the dynamic inference pipeline requires (1) faithful tracking of relevant elements of the tissue through time, (2) an underlying model for the stress at each membrane, and finally, (3) knowledge of the relationship between the scales involved in the process under study.

To the authors’ knowledge, three different methods have been proposed to deal with time series of data: DLITE [67], video force microscopy (VFM) [57], and ForSys [66]. Only DLITE and ForSys are currently available. In the following sections, we explore the virtues and limitations of these three methods.

### DLITE: time series as an initial condition

**Figure 5.**
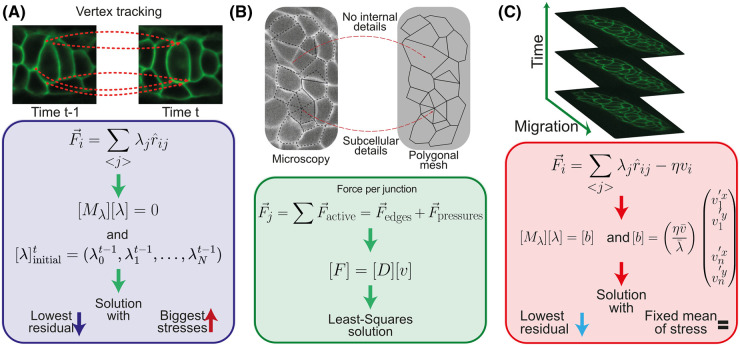
Dynamic stress inference. (**A**) DLITE is not a dynamic method *per se*. It tracks each junction of the system through time and assumes that the force (${\vec{F}}_{i}$) is in the direction of the membrane ($\hat{r_{ij}}$) and scaled by the membranes’ stress (λ). The system is compiled in a geometrical matrix ($\left[M_{\lambda }\right]$) and solved using the solution at the previous time point as an initial guess for the numerical algorithm. (**B**) VFM uses a Finite Element Mesh to evaluate the system's movements. The mesh does not need to coincide with the cellular details. Stresses and pressures are intertwined in the equations. The system to solve establishes a relationship between the force ([F]) and the velocity of the nodes ([v]) through a damping matrix ([D]). (**C**) ForSys solves the equations by considering each junction's velocity (incorporated in the [b] matrix), mediated by the scale parameter ($\eta \bar{v}/\bar{\lambda }$), which can be interpreted as the reciprocal of the Weissenberg number. Through this number, the relationship between viscous and elastic scales must be tuned. The neuromast's membranes shown in panel B and the primordium's in panels A and C were tagged using claudnb:lyn-EGFP.

The use of movement information, also reviewed in [35], allows tracking of the nodes through time, dealing with the first issue presented at the start of the previous section. DLITE (Dynamic Local Intercellular Tension Estimation) [67] assumes mechanical equilibrium at each junction (eqn 1), similar to CellFIT [60]. DLITE takes membrane tensions in the direction of the edge joining the junction, with no additional terms (eqn 2). Moreover, it uses a regularizer that penalizes small stress values to avoid the null solution. Though time series tracking of tissues is involved, as Roffay et al. [35] pointed out, this is not a dynamic inference method *per se*. In fact, DLITE only tracks all nodes, edges, and cells through time to use the inferred solutions of the previous time as an initial guess for the current frame (Figure 5A). Importantly, the authors show that this is sufficient to improve performance on in silico data and maintains better robustness over time than CellFIT [67]. Moreover, unlike CellFIT, DLITE is an open-source project, allowing users to examine the details of its implementation.

### VFM: dynamic stress inference in a mesh

Brodland et al. [57] reported the first dynamical stress inference of tissues to study the mechanics of ventral furrow invagination in Drosophila. It was called VFM and previously cinemechanometry [58]. They developed this method in a series of papers spanning more than fifteen years [86–89]. VFM successfully identified stresses during ventral furrow failure due to reduced myosin II activity [57]. The authors built a finite element mesh over the tissue of interest's active landmarks (Figure 5B). This meshing need not correspond precisely with all the cell's hallmarks, namely the cell membranes, and could have subcellular details. Brodland et al. chose the mesh so that its nodes correspond with the elements they categorize as active. Therefore, it could be the case that the polygons enclosed by the mesh are not cells but rather subcellular domains (Figure 5B, upper panel).

They assumed that all passive elements’ contributions could be subsumed in generating an effective viscosity [57]. In this model, the forces of the active components ($f_{t}^{A}$) are equal to the velocity of the nodes ($v_{t}$), mediated by a damping matrix ($D_{t}$) with viscosity information, so at each time-point *t*

9 $$f_{t}^{A}=D_{t}v_{t}$$

In contrast with the previously mentioned CellFIT method [60], VFM calculates the contribution of each force from a geometrical matrix using a straight-edge approach, which combines both the stresses and the pressures. Importantly, unlike DLITE and ForSys (discussed in the next section), VFM is not currently available.

### ForSys: dynamic inference on the vertices

Most recently, we proposed the ForSys method, which builds upon the advancements in the field and integrates them into an open-source pipeline [66]. This software allows Static and Dynamic Stress Inference in curved geometries, which is achieved by fitting a circle to the segmented edges [66], as in CellFIT and DLITE [60,67].

ForSys benefits from the viscous forces prevalent in the tissue and modified eqn 1 to include a damping term as

10 $$\underset{j}{\sum }\lambda_{j}{\vec{r^{i}}}_{j}=\eta \vec{v^{i}}$$

where η is the damping constant and $\vec{v^{i}}$ the velocity of the vertex *i*. Implementing an overdamped regime mathematically transforms the system into an inhomogeneous system of equations, as the [*B*] term present in equs 3, 6, and 8 is now different from the null matrix. This matrix now includes the velocity components of all junctions in the calculation. An immediate advantage of this method is that it is no longer necessary to avoid the case where all forces in the junction are zero.

As shown in eqn 1, the left-hand side has dimensions given by the stress λ. However, in Dynamic Stress Inference, while the left-hand side still has this dimension, the right-hand side now has units of $\eta \vec{v}$, as shown in eqn 10. Thus, correctly determining the scales involved is crucial, as pointed out at the beginning of the ‘Dynamic Stress Inference’ section. eqn 10 can be transformed into a non-dimensional form with one free parameter proportional to the Weissenberg number's reciprocal [66]. This well-known non-dimensional quantity relates the elastic and viscous forces through a reference stress $\bar{\lambda }$, velocity $\bar{v}$, and damping coefficient η as

11 $$\frac{1}{W_{i}}=\frac{\eta \bar{v}}{\bar{\lambda }}$$

Therefore, the force at each junction is

12 $$\underset{j}{\sum }\lambda_{j}^{\prime }{\vec{r^{i}}}_{j}=\left(\frac{\eta \bar{v}}{\bar{\lambda }}\right)\;\vec{{v^{\prime }}^{i}}$$

where $\;\lambda_{j}^{\prime }=\lambda_{j}/\bar{\lambda }$ and $\;\vec{{v^{\prime }}^{i}}=\vec{v^{i}}/\bar{v}$. The scale relation introduced by this work might extend beyond the particularities of ForSys and could be used in other dynamic measurements, such as in Transverse Fluctuation (TFlux) [90,91].

When tested using *in silico* tissue movies, this method outperforms other software when the junctions move significantly and negligibly [66]. Furthermore, this method opened a window into using force inference technology in migratory structures. When applied to the mobile Zebrafish lateral line primordium, the method detected zones of high pressure/tension that indicate the presence of rosettes predating the separation of the neuromast organs [66].

## Outlook

As with any technique, its falsifiability is an important aspect of stress inference. The standard for validating the inference engine in static and dynamic modalities is based on *in silico* validations, often generated using a cell-based computational model such as the Vertex model [45,67,69,92]. Various experimental validations can be performed for static inference by fluorescent measurements, such as with myosin intensity [59], antibodies staining [93,94], and flipper probes [95], or through direct manipulation with methods including laser ablation [45,59] and atomic force microscopy (AFM). Complementary to hydrostatic pressure measurement [96,97], methods for osmotic pressure determination have recently emerged [55]. Pressure inference cannot distinguish between these contributing factors; however, we expect the inferred pressure to be dominated by the hydrostatic component, as osmotic pressure differences equilibrate rapidly across the tissue. In contrast, experimental validation of dynamic inference by ground truth generation is rather limited, as methods such as laser ablation or AFM irreversibly change the state of a tissue and are likely to disrupt or alter the processes of interest. Therefore, dynamic stress inference is typically calibrated using computational model simulations.

Thanks to advances in image segmentation, static stress inference can be performed in 3D, as initially reported by Brodland and colleagues [98] and more recently by the Turlier lab [92]. Incorporating the third dimension into dynamic inference will be a significant leap forward. Reliable 3D stress maps that could be used to validate an inference technique are experimentally challenging to produce, especially if the maps need to be time-dependent.

A desirable feature of future stress inference techniques is to couple the inference engine with the computational packages for simulating tissues using cell-based models that rely on mechanical information, such as the vertex model. This model requires the correct parameterization of the line tensions needed to simulate tissues that recapitulate the phenomena they are intended to describe [63]. In this way, the intercellular stresses obtained by stress inference could be used to parameterize a model and generate predictions embodied in model simulations.

Stress inference can uncover mechanical features of systems in various settings. It has been successfully applied to investigate cell division [40], organogenesis [66], and cell-type-specific mechanical anisotropies [39,66,99]. We expect that stress inference will expand its applications to fields such as immunology [100] and cancer biology [101,102] in the coming years, which could greatly benefit from this technique.

While stress and pressure inference allows the determination of local intracellular pressure and intercellular stress, one might expect that this information could be integrated to provide a whole tissue description of the mechanical state of the system. Recently, it was proposed that local and tissue-level mechanical information could be combined with spatial omics [103]. This will allow a deeper understanding of the connection between the mesoscopic scale and the molecular details of the interactions.

Even though a machine-learning approach has recently been used to infer forces from cytoskeletal protein distributions [104], a new avenue to explore could be using machine-learning algorithms to infer stresses. This would involve curating microscopy images and feeding them into a neural network, which in turn could learn to predict stress distribution for a given topology. To our knowledge, this exciting possibility has not been explored even in the static inference formalism.

## Perspectives

- Dynamic stress inference provides a first computational approach to determine the mechanical state of tissues and generate predictions that can guide future experiments. This family of inexpensive tools can advance the study of tissues in development and regeneration.
- While static stress inference from microscopy images allows the characterization of a tissue's mechanical state at a single point in time, dynamic stress inference from video microscopy represents the full spatiotemporal distribution of mechanical stresses experienced by the tissue.
- A next logical step will be to combine dynamic inference with 3D geometric inference to ultimately generate 4D stress inference. Another important step will be the coupling of inference with cell-based models, which will allow direct testing of these models in biologically relevant geometries. Finally, machine learning algorithms that allow the training of networks on specific organisms may prove helpful in recognizing specific patterns of stress distributions.

## Data Availability

The *in silico* image where generated using seapipy and ForSys. The seapipy codebase is available on GitHub at https://github.com/borgesaugusto/seapipy and Zenodo [105]. ForSys codebase is available on GitHub at https://github.com/borgesaugusto/forsys and Zenodo [106].

## Footnotes

^1^
In the present review, we will not address continuous methods for stress inference. They include methods that rely on hydrodynamic calculations to convert cell displacements into stress maps [33–36] or using displacement from photopatterned substrates, such as Traction Force Microscopy [37,38].

## Open Access

Open access for this article was enabled by the participation of University of Nottingham in an all-inclusive *Read & Publish* agreement with Portland Press and the Biochemical Society under a transformative agreement with JISC.

## Abbreviations

AFM: atomic force microscopy
DLITE: Dynamic Local Intercellular Tension Estimation
VFM: video force microscopy
