# Supplementary material for: Peeking into the future: inferring mechanics in dynamical tissues
Source: Biochem Soc Trans. 2024 Dec 10;52(6):2579–92. doi: 10.1042/BST20230225 (PMC11668348; doi:10.1042/BST20230225)
Supplement: Supplementary Material 1 [file BST-52-2579-s1.pdf]

A B C

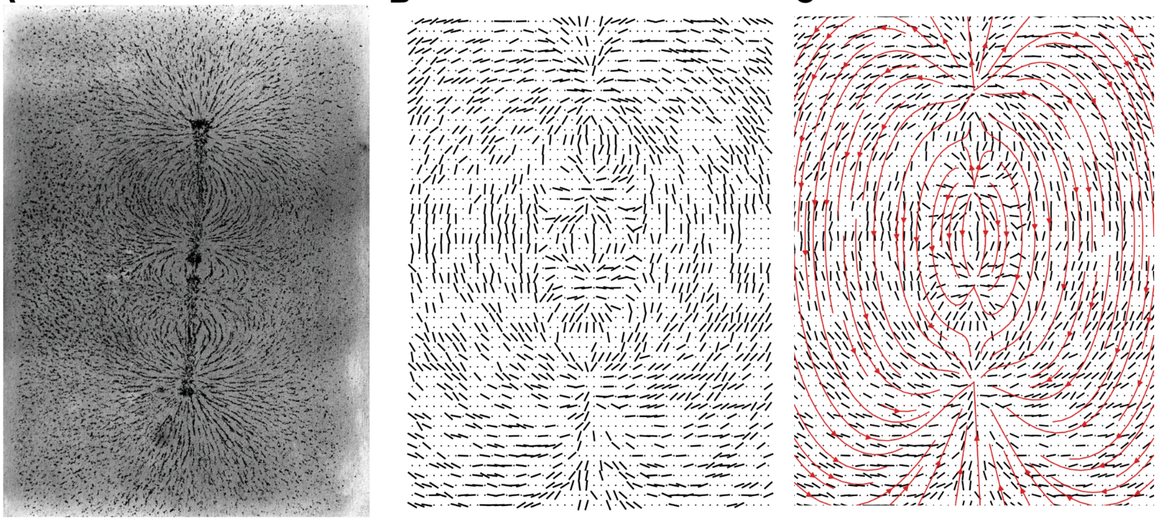

Supplementary Figure1. Stress as a patterning guide. Iron filings are arranged by magnetic fields (Credit: M0000164: Michael Faraday's iron filings diagram. Wellcome Library has provided this material; GB CC BY 4.0). **(A)**, a behavior that can be modeled computationally **(B)**. This arrangement is a consequence of the underlying field **(C)**. To create this, we simulated the magnetic field of 4 charges at different positions along the y-axis. The black lines represent the direction of the field with a Gaussian noise of 10 degrees; only around 50 % of them are shown. Similarly, tissues are also shaped according to the mechanical state to which they are subjected.
